# A retrospective cohort study evaluating the predictive value of urinary L-FABP combined with the SOFA score for assessing COVID-19 severity

**DOI:** 10.1371/journal.pone.0331558

**Published:** 2025-09-11

**Authors:** Kanako Terakawa, Daisuke Katagiri, Yusuke Asai, Masahiro Ishikane, Norio Ohmagari, Minami Suzuki, Masayuki Hojo, Hideki Takano, Katsushi Tokunaga, Takeshi Sugaya, Eisei Noiri

**Affiliations:** 1 Department of Nephrology, National Center for Global Health and Medicine, Japan Institute for Health Security, Tokyo, Japan; 2 Division of Nephrology and Endocrinology, University of Tokyo Graduate School of Medicine, Tokyo, Japan; 3 Center for Clinical Sciences, Japan Institute for Health Security, Tokyo, Japan; 4 Disease Control and Prevention Center, National Center for Global Health and Medicine, Japan Institute for Health Security, Tokyo, Japan; 5 Department of Respiratory Medicine, National Center for Global Health and Medicine, Japan Institute for Health Security, Tokyo, Japan; 6 Central Biobank, National Center Biobank Network, National Center for Global Health and Medicine, Japan Institute for Health Security, Tokyo, Japan; 7 Timewell Medical Co., Ltd., Tokyo, Japan; Mahidol University, Faculty of Tropical Medicine, THAILAND

## Abstract

Although the symptoms of Coronavirus Disease 2019 (COVID-19) omicron strains are generally mild, some individuals with initially mild symptoms later required supplemental oxygen or hospitalization, while others died, highlighting the importance of rapid diagnosis and early treatment for such patients. Therefore, this study evaluated the benefit of a combined approach to identify individuals at risk of severe illness due to COVID-19 using urinary L-type fatty acid-binding protein (L-FABP) levels and the Sequential Organ Failure Assessment (SOFA) scores calculated from blood tests for pre-admission screening of patients with COVID-19. L-FABP, a non-invasive urine biomarker, and the SOFA score, an established method for assessing organ failure severity, were evaluated in conjunction with patient data collected from 842 individuals admitted to a hospital in Tokyo, Japan. The combined approach demonstrated a higher accuracy in identifying patients at risk of severe illness compared to the L-FABP levels or SOFA scores alone. Thus, the results suggest that a two-tiered screening process, utilizing measurement of L-FABP levels as an initial rapid test, followed by SOFA score assessment for high-risk patients, results in efficient pre-admission screening and improved patient management. In conclusion, this study underscores the potential of combining L-FABP levels and SOFA scores for optimizing patient care during the ongoing COVID-19 pandemic, emphasizing the need for further validation and refinement of the predictive models.

## Introduction

In March 2020, the World Health Organization (WHO) declared the outbreak of coronavirus disease 2019 (COVID-19), caused by Severe Acute Respiratory Syndrome Coronavirus 2 (SARS-CoV-2), a global pandemic. Since its emergence, the virus has spread rapidly across the world, giving rise to numerous variants. Consequently, the clinical presentation of COVID-19 has become widely recognized among healthcare professionals. Currently, emergent Omicron sublineages—each bearing a distinct constellation of mutations from the ancestral Omicron variant—predominate the course of the pandemic. Although the symptoms of Omicron strains are generally mild, some individuals experience severe illness and even fatality. For instance, some patients, especially high-risk individuals, with initially mild symptoms later required supplemental oxygen or hospitalization, while others died. This highlights the critical need for predicting the severity of illness in SARS-CoV-2-positive patients, particularly when the strain on healthcare systems is intensified by an increasing number of such patients.

Consequently, we previously described a noninvasive approach for predicting COVID-19 severity using the urine biomarker L-type fatty acid binding protein (L-FABP) [1]. Early measurement of urinary L-FABP levels facilitates the identification of patients prone to severe COVID-19 outcomes [2]. However, our study was limited by its small sample size (N = 58). Although another study assessed L-FABP levels in patients with COVID-19, they were not assessed in combination with other previously studied risk factors, such as the Sequential Organ Failure Assessment (SOFA) scores, lactate dehydrogenase level, or D-dimer level [3,4].

Therefore, in this study, we aimed to assess the benefit of a combination approach, integrating L-FABP levels and SOFA scores for screening patients with COVID-19, to identify individuals at risk of severe illness and refine risk prediction strategies.

## Materials and methods

### Patient population

This single-center retrospective observational study included patients who tested positive for COVID-19 and were admitted to the National Center for Global Health and Medicine between January 29, 2020, and April 6, 2022. Patients were screened for eligibility based on a positive real-time reverse transcriptase-polymerase chain reaction (RT-PCR) assay for SARS-CoV-2 using nasal swabs. Patients who met the following criteria were excluded: 1) patients who could not provide consent, 2) those on maintenance dialysis, 3) those younger than 18 years old, 4) whose L-FABP levels or SOFA scores could not be evaluated, and 5) whose urine specimens could not be collected within 3 days of admission and 10 days of symptom onset. Patient demographic data, clinical symptoms, and radiographic and laboratory findings were collected. Specimen analysis was conducted within 3 business days of hospitalization, adhering to previously established criteria and procedures [5].

### Definitions and biomarker measurement

The primary aim of this study was to evaluate whether urinary L-FABP levels measured at admission in patients with COVID-19 can predict disease severity on day 7. We used a previously described method to assess COVID-19 severity [5]. Patients requiring intensive care interventions, such as mechanical ventilation, or those whose outcome was death were categorized as “severe,” those receiving oxygen therapy but not requiring ventilation were classified as “moderate,” while the remainder were deemed “mild.” Deceased patients were classified as “severe” in the final outcome during data aggregation. However, their classifications at the time of admission and on day 7 were based on the predefined criteria while the patients were still alive, and therefore, they were not necessarily categorized as “severe” at those time points. Deaths were included regardless of whether they were due to direct respiratory failure caused by COVID-19 or from other causes such as frailty and deterioration during prolonged hospitalization, particularly among older patients. Urine samples were collected at two time points – within three calendar days following admission (from now on, referred to as “on admission”) and on day 7 – to measure urine L-FABP levels. L-FABP levels were determined using a latex-enhanced turbidimetric immunoassay as per the manufacturer’s instructions (Denka Seiken and Sekisui Medical, Tokyo, Japan). Henceforth, “L-FABP levels” refers to urinary L-FABP levels adjusted for urine creatinine concentration. Adjustment for creatinine levels in urinary studies is a well-established method for normalizing different urine concentrations [6,7]. SOFA scores are based on six scores ranging from 0–4, with one point assigned for symptoms or signs in the respiratory, circulatory, hepatic, coagulation, renal, and nervous systems including blood tests, with higher scores reflecting worse organ dysfunction [8].

### Statistical analysis

Baseline characteristics and clinical findings of the patients were examined upon admission. Continuous variables are presented as medians and interquartile ranges (IQRs), and categorical variables are presented as the absolute number of cases and their respective percentages. Receiver operating characteristic (ROC) curve analysis was performed to evaluate the predictive performance of on-admission urinary L-FABP levels and SOFA scores. Spearman’s correlation coefficient between urinary L-FABP levels and SOFA scores on admission was calculated using the *cor.test* function. Multivariable logistic regression analysis was performed to identify factors predicting severe and severe/moderate cases. Clinically relevant variables were investigated by the study investigators. In addition to L-FABP and SOFA score admission, the variables age, sex, BMI, smoking history, hypertension, diabetes mellitus, hyperlipidemia, chronic obstructive pulmonary disease, chronic kidney disease, asthma, and days since onset to admission were selected, and their multicollinearity was assessed by variance inflation factor (VIF). When multicollinearity is observed (VIF > 3), variables more associated with increased risk are selected (however, no variables showed obvious collinearity in the analysis). When the number of cases is limited and all the relevant variables cannot be employed, univariate logistic regression analysis is carried out. The logistic regression analyses were performed using *glm* function. The area under the curve (AUC) and its 95% confidence interval were calculated from the ROC curve, and the cutoff values, sensitivity, and specificity were determined using Youden’s index. Pairwise comparisons of AUCs were performed using the DeLong method. The *roc, ci,* and *roc.test* functions in the *pROC* package in R were used for ROC analysis. All statistical analyses were performed using R statistical software version 4.3.1.

### Ethics approval

The study protocol was approved by our Institutional Review Board (NCGM-G-003616), and written informed consent was obtained from each patient before enrollment. The study was performed in accordance with the Declaration of Helsinki and with the relevant guidelines and regulations (ClinicalTrials.gov number: NCT04681040). On July 1, 2024, we accessed the data for research purposes. During or after data collection, the authors did not have access to information that could identify individual participants.

## Results

### Patient characteristics and clinical courses

**Fig 1:**
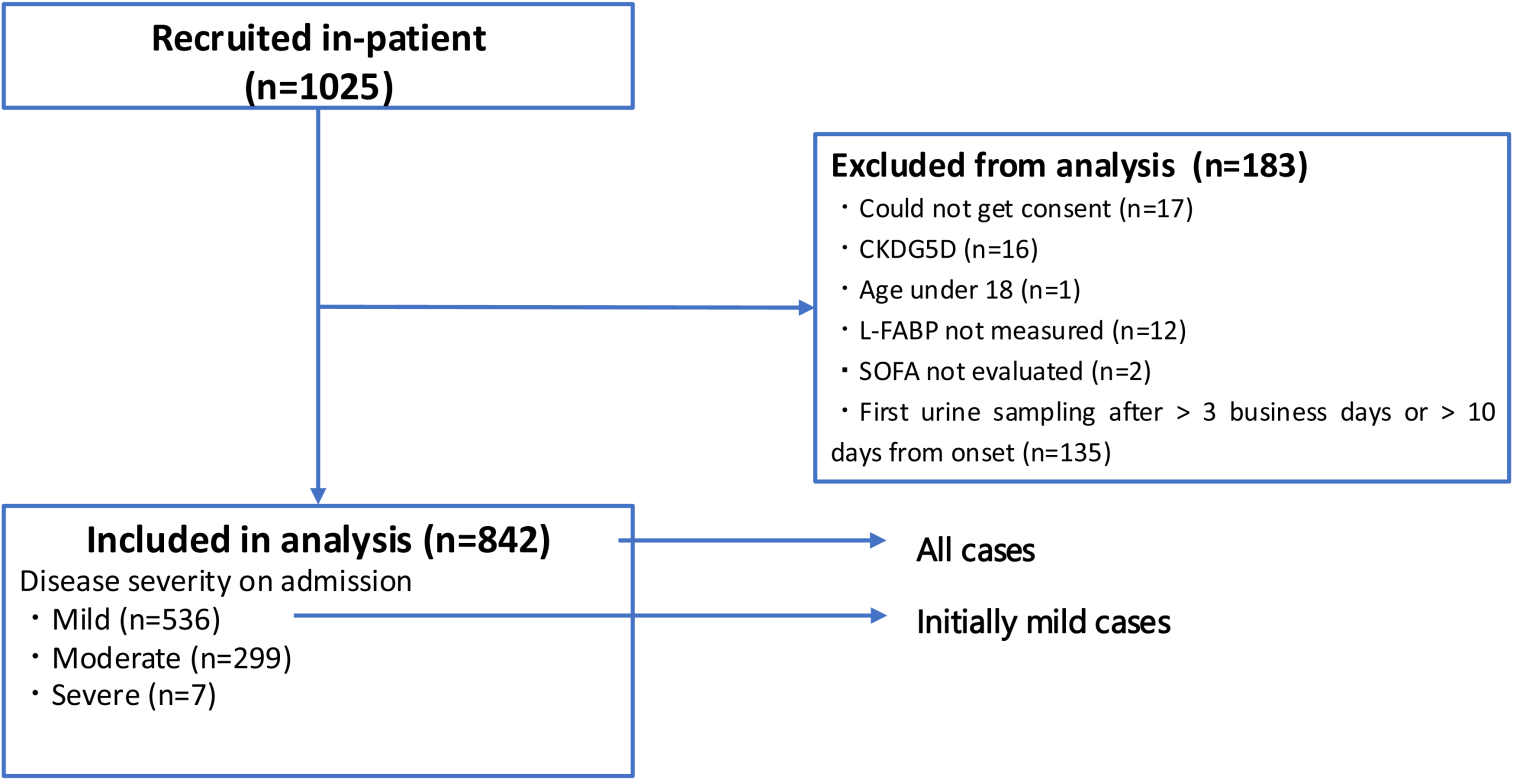
Flowchart of patient enrollment.

A total of 1025 participants were initially recruited. After excluding those who did not meet the inclusion criteria, 842 patients were ultimately included in the study. Patient characteristics including comorbidities and time from symptom onset to admission are summarized in Table 1. A flowchart of patient enrollment is shown in Fig 1. All patients underwent severity-related interventions during hospitalization. At the time of admission, 536 of the 842 patients were categorized as mild, 299 as moderate, and seven as severe. Thirty-two patients died during hospitalization. The transition in disease severity between admission and day 7, including the number of patients progressing or improving in severity, is summarized in Table 2. On Day 7, 55 patients who were initially categorized as moderate on admission improved to the mild category. Additionally, the number of patients classified as severe increased to 34 compared to that on admission. Among the patients who were initially categorized as mild, 457 remained mild category, whereas 77 became moderate, and two developed severe conditions. Among patients who were initially classified as moderate, 219 remained moderate, while 25 progressed to severe conditions.

**Table 1:** Patient characteristics.

Characteristics	Values
No. of patients	842
Age – years (Median (IQR))	53 (41–69)
Sex – no. (%)	Male 570 (67.7): Female 272 (32.3)
Overweight (BMI > 25) – no. (%)	352 (43.5)
Smoking – no. (%)	349 (41.4)
Hypertension – no. (%)	204 (24.2)
Diabetes Mellitus – no. (%)	132 (15.7)
Hyperlipidemia – no. (%)	108 (12.8)
Chronic Obstructive Pulmonary Disease – no. (%)	10 (1.2)
Chronic Kidney Disease – no. (%)	18 (2.1)
Asthma – no. (%)	59 (7.0)
Days since onset to admission (Median (IQR))	5 [3–7]

**Table 2:** Severity of disease.

		at day 7			
		Mild	Moderate	Severe	All
On admission	Mild	457	77	2	536
	Moderate	55	219	25	299
	Severe	0	0	7	7
	All	512	296	34	842

In total, 1025 participants were recruited. After excluding patients that failed to meet the inclusion criteria, 842 patients were included in the study. Receiver operating characteristic (ROC) curve analysis was performed to evaluate the performance of urine L-FABP and SOFA scores upon admission. Abbreviations: L-FABP, L-type fatty acid-binding protein; SOFA, Sequential Organ Failure Assessment; CKDG5D, Patients with end-stage kidney disease who are on dialysis.

### Identifying high-risk COVID-19 patients using L-FABP levels, SOFA scores, and their combination

**Fig 2:**
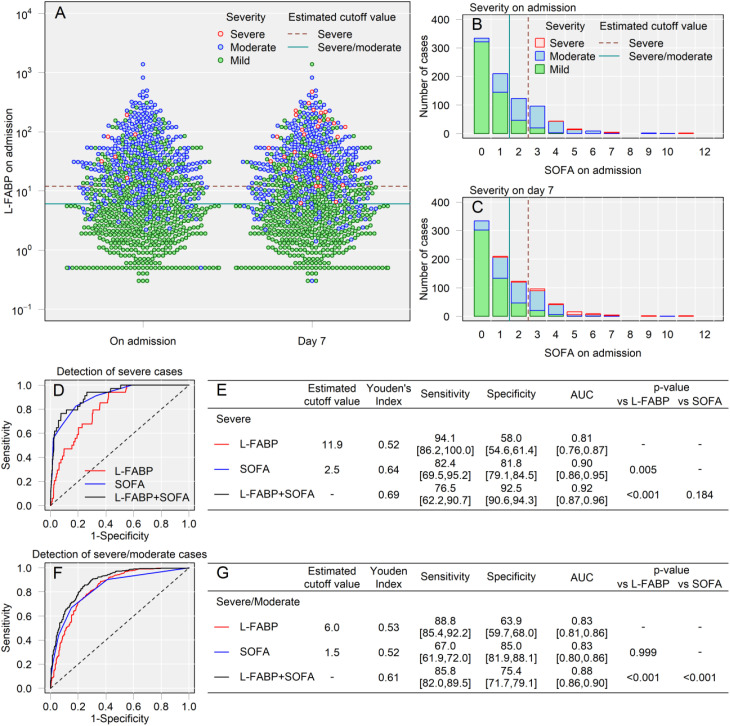
Disease severity progression and biomarkers. **A.** Visualization of disease severity and biomarker levels. Each dot represents an individual patient, with color indicating disease severity (green = mild, blue = moderate, red = severe). The green and red lines denote the estimated L-FABP cutoff values for identifying moderate/severe and severe cases, respectively. **B and C.** The x-axis indicates the SOFA score on admission for both panels. Panel B shows the severity on admission, while Panel C shows the severity on day 7. The severity is divided by color, with red indicating severe disease, blue indicating moderate disease, and green indicating mild disease. **D and F.** Receiver operating characteristic (ROC) curves and area under the curve (AUC) analyses for severity classification. ROC curves for L-FABP levels, SOFA scores, and their combination (L-FABP+SOFA) are shown for the detection of severe (D) and moderate/severe (F) cases. **E and G.** Summary tables presenting sensitivity, specificity, AUC, and estimated cutoff values for severity classification. Panel E corresponds to severe case detection, while Panel G pertains to moderate/severe case detection.

We visualized the course of disease severity by plotting the L-FABP levels of all analyzed patients at two time points: admission and day 7 (Fig 2A). The L-FABP levels were plotted in the same relative position on the corresponding graphs on both measurement days. The colors of the symbols indicate the severity of disease at the corresponding point in time. Green indicates mild cases that did not require oxygen administration, blue indicates moderate cases that required oxygen administration, and red indicates severe cases that required ventilation. Several patients with initially high L-FABP levels upon admission transitioned from green to blue or blue to red after seven days, indicating progressive disease severity. Similarly, we visualized the trajectory of disease severity on admission and on day 7 by comparing the distribution of case severity with the SOFA scores (Fig 2B, C). Patients with high SOFA scores at admission were more likely to exhibit severe disease after seven days.

ROC curve analysis was performed to assess the effectiveness of various combinations of urinary L-FABP and SOFA scores in distinguishing clinically severe and severe/moderate cases (Fig 2D, F). Urinary L-FABP levels demonstrated high sensitivity (94.1%) in identifying severe cases at a cutoff value of 11.9, outperforming the SOFA score and indicating its potential as an effective screening tool (Fig 2D, E). Fig 2 presents the AUC comparisons among three groups: L-FABP vs. SOFA, L-FABP vs. L-FABP+SOFA, and SOFA vs. L-FABP+SOFA, for both severe and severe/moderate case detection (Fig 2E, G). Combining L-FABP levels (AUC 0.81) with SOFA scores (AUC 0.90) increased the AUC to 0.92 for severe case detection. However, this improvement was not statistically significant when compared to the SOFA score alone (Fig. 2E). In contrast, the combination of L-FABP levels and SOFA scores was significantly more effective than either marker alone for detecting severe or moderate cases (Fig 2G).

### SOFA score cutoff value after pre-screening with L-FABP

**Fig 3:**
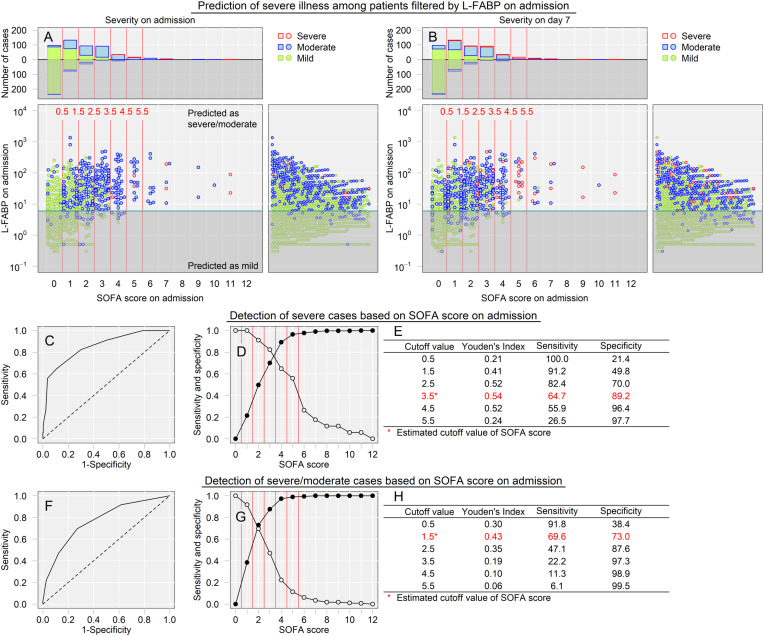
Prediction of severe cases among patients filtered by L-FABP on admission. **A.** Each dot represents a patient, with the color indicating disease severity (green = mild, blue = moderate, red = severe). The green line shows the estimated cutoff value of L-FABP levels to detect severe cases. **B.** Each dot represents a patient, with the color indicating disease severity on day 7 (green = mild, blue = moderate, red = severe). The green line indicates the estimated cutoff value of L-FABP levels to detect severe cases. **C.** Receiver operating characteristic (ROC) curve analysis for detecting severe cases based on the SOFA scores at the time of hospital admission. **D.** This panel shows the sensitivity (white dots) and specificity (black dots) of different SOFA score cutoff values for detecting severe cases. **E.** The estimated cutoff value of the SOFA score to detect severe cases is 3.5, with a sensitivity of 64.7% and a specificity of 89.2%. **F.** ROC curve analysis for detecting severe/moderate cases based on the SOFA scores at the time of hospital admission. **G.** This panel shows the sensitivity (white dots) and specificity (black dots) of different SOFA score cutoff values for detecting severe/moderate cases. H. The estimated cutoff value of the SOFA score to detect severe/moderate cases is 1.5, with a sensitivity of 69.6% and a specificity of 73.0%.

We then assessed the predictive power of urinary L-FABP levels at admission to identify severe cases (Fig 3). Fig 3A depicts the data upon admission, with each data point representing a patient color-coded according to the disease severity. Green, blue, and red points indicate mild, moderate, and severe disease, respectively. The green line represents the identified L-FABP cutoff value (6.0 μg/gCr) for detecting moderate and severe disease, as shown in Fig 2. The L-FABP levels and SOFA scores are plotted on the vertical and horizontal axes, respectively. We observed that most patients whose SOFA values on admission were initially above the cut-off (green line) for L-FABP levels and had elevated SOFA scores and later transitioned to a more severe status, as shown by the colors by comparing this graph to Fig 3B. Thus, the patients tended to develop an increased oxygen demand or required ventilatory support with huge clinical impact, indicating progression to critical illness.

Additionally, only considering patients with elevated L-FABP levels upon admission revealed that those with higher SOFA scores were at an increased risk of developing severe disease. Therefore, we further evaluated the optimal SOFA score for predicting patients whose condition was likely to worsen. Fig 3C, D. shows the sensitivity (represented by white dots) and specificity (represented by black dots) for various SOFA score cutoff values in detecting severe cases. Fig 3E indicates that the identified optimal cutoff value of SOFA scores for detecting potentially severe cases was 3.5, achieving a sensitivity of 64.7% and a specificity of 89.2%. Whereas Fig 3F, G and 3H show that the estimated best cutoff value of the SOFA score was 1.5, with a sensitivity of 69.6% and specificity of 73.0% to detect combined potentially severe and moderate cases. Supplementary S1 Fig presents the results of logistic regression analyses for identifying severe cases (Panel B) and combined severe/moderate cases (Panel C).

Both L-FABP and the SOFA score were found to be valuable markers for detecting these cases, either independently or in a complementary manner.

## Discussion

This study aimed to establish a practical pre-admission screening method to identify patients with COVID-19 at risk of hospitalization and develop a tool for predicting severe cases by day 7 of hospitalization. Our results showed that the combination of L-FABP levels and SOFA scores had the highest diagnostic accuracy (AUC = 0.918) for identifying patients at risk of severe illness.

During the COVID-19 pandemic, the Japanese healthcare system was stressed, especially during the fourth and fifth waves (April-September, 2021), resulting in several patients with mild-to-moderate COVID-19 who could no longer be transferred to specialized hospitals even if they deteriorated, and patients at home or in designated lodging facilities could not be admitted to any hospital. Unfortunately, a significant number of these patients died at home during this period. Although bed capacity was insufficient, the situation could have been better managed if proper screening was available to maximize bed utilization [9].

We previously reported that patients with severe COVID-19 exhibit elevated urinary L-FABP levels upon admission [1], consistent with another study [10], which demonstrated that L-FABP levels in severe COVID-19 tend to increase shortly after symptom onset and remain elevated. L-FABP is predominantly localized in the cytoplasm of the proximal tubular cells in human kidneys and has a molecular weight of 14–15 kDa. L-FABP plays a pivotal role in binding free fatty acids within the cytoplasm and shuttling them to mitochondria and peroxisomes, thereby facilitating beta-oxidation to support energy production and maintain cellular homeostasis [11]. Additionally, urinary L-FABP is a highly sensitive and specific biomarker for detecting ischemic kidney injury [12] because of a specific DNA sequence, the hypoxia response element, which is located upstream of the *FABP1* gene. This is similar to the erythropoietin gene, which is involved in oxygen sensitivity. Erythropoietin is produced in the peritubular capillary endothelial cells of the kidney while L-FABP is produced in the proximal tubular cells of the same region. Moreover, the kidneys are highly vascularized organs that receive approximately 20% of the cardiac output and are crucial for maintaining systemic water and solute balance. Thus, they are well suited for hypoxia sensing. Consequently, L-FABP expression increases in response to hypoxia, suggesting that hypoxic conditions in COVID-19 that do not cause acute kidney injury can elevate urinary L-FABP levels [10,13]. Although L-FABP is an established biomarker of acute kidney injury, because it reflects tubular damage [14,15], it has also been utilized to assess overall illness severity in patients [16,17]. Mascle et al. recently reported that disease severity was higher in a group of patients with COVID-19 with higher urinary L-FABP levels [18]. Furthermore, unlike common blood tests, which are invasive and require blood collection, the measurement of urinary L-FABP offers a non-invasive alternative that can be performed with minimal patient discomfort.

Meanwhile, SOFA scores are used to evaluate the severity and prognosis of organ failure in critically ill patients in the intensive care unit (ICU). Developed in 1994, they have been widely used to assess sepsis severity and multi-organ failure [19,20]. Additionally, a previous study suggested that an elevated SOFA score may be an independent risk factor for in-hospital death and could be used to assess the severity and prognosis of COVID-19 [3].

Notably, we used the SOFA score rather than the quick SOFA (qSOFA) score in this study. The qSOFA score is a shorter version of the SOFA score, requires fewer input measurements, and is typically used to screen for sepsis in patients with suspected infection, which puts them at a high risk of in-hospital mortality outside the ICU [20]. However, an increase in the SOFA score by two or more units in a cohort admitted to the ICU with suspected infection demonstrated superior prognostic accuracy for in-hospital mortality compared to the systemic inflammatory response syndrome criteria and the qSOFA score [21]. This suggests that the qSOFA score may have limited accuracy in predicting mortality compared with the SOFA score. Additionally, a study demonstrated that the SOFA score was superior to the qSOFA score, particularly as a risk stratification tool at admission for critically ill patients with COVID-19 [22]. Moreover, blood test results are required to calculate the SOFA score, along with vital signs such as blood pressure, respiratory rate, and mental status, whereas the qSOFA score only requires vital signs. This difference in the amount of required data likely explains the difference between the effectiveness of the SOFA and qSOFA scores regarding risk prediction of COVID-19 disease progression.

In the present study, L-FABP levels showed high sensitivity for detecting patients at risk of developing severe COVID-19. Specifically, 94% of severe cases and 89% of severe or moderate cases were detected using L-FABP levels. Biomarkers typically require high sensitivity (≥ 90%) in international target product profiles to maximize true positive rates and be useful as a first-line risk assessment tool [23]. Accordingly, in this cohort, urinary L-FABP at admission achieved a sensitivity of 89% in detecting patients who subsequently developed severe disease, approaching this threshold value and supporting its potential as an early triage tool. Moreover, urine testing has several key advantages over blood testing, establishing it as the superior choice in various clinical settings. First, urine tests are non-invasive, as they do not require needle insertion, making the process more comfortable and less stressful, and imposing less burden on patients. Second, urine tests offer improved safety. The risk of infection is eliminated because blood withdrawal is not required, thereby providing a safer option for patients and healthcare providers. Third, urinary testing is highly convenient as it is simple to perform and does not require specialized skills or equipment, allowing for easy implementation in diverse settings, including outpatient clinics and homes. Finally, urine tests are ideally suited for repeatability. Their ease and safety allow for frequent monitoring, enabling repeated assessments over time to track a patient’s condition or treatment progress without causing undue stress. Collectively, these advantages make urine testing a preferable option to blood testing, particularly when frequent, safe, and accessible monitoring is essential. Thus, the ease of administering a urine test makes L-FABP an ideal first step in screening severe COVID-19 cases, particularly in settings where rapid and non-invasive testing is crucial.

Therefore, we used a non-invasive L-FABP assay with established cutoff values as the first step of our proposed two-step pre-admission screening process to identify patients at risk of severe or moderate COVID-19. This rapid test, which does not require blood collection, can be performed at outpatient clinics, adult care facilities, or emergency departments to identify patients at low risk of clinical deterioration. Individuals with low L-FABP levels in our cohort infrequently progressed to severe illness. However, they should continue to undergo routine clinical monitoring, and prompt escalation of care—including repeat biomarker assessment when clinically indicated—should be initiated if their condition worsens. Subsequently, a blood test can be performed to determine the SOFA score for patients identified as high risk based on the L-FABP assay. This score, which includes vital signs, such as blood pressure, respiratory rate, and mental status, can further assess the need for hospital admission and oxygen supplementation.

Thus, we propose a sequential two-tiered screening strategy. First, the urinary L-FABP test is performed to identify potential high-risk individuals, allowing low-risk patients to avoid unnecessary admission. Second, the SOFA score refines risk stratification for patients with elevated L-FABP levels by pinpointing those who are most likely to worsen and require hospitalization, thereby guiding treatment and resource allocation. As illustrated in Fig 2F and 2G, no significant difference was observed between L-FABP levels and SOFA scores alone in detecting moderate/severe cases. However, the combination of L-FABP and SOFA scores demonstrated a significantly better performance than either marker alone. Specifically, combining the L-FABP levels with SOFA scores increased the specificity for identifying patients at risk (i.e., fewer false positives) compared to using L-FABP levels alone, while still maintaining a high sensitivity (Fig 2G).

While research has focused on cytokine storms in COVID-19, microvascular dysfunction caused by hypercoagulation may offer a more accurate picture of its pathophysiology [24]. Most patients with COVID-19 experience mild or no symptoms; however, severe cases can develop complications such as thromboembolism and respiratory failure. Arteries and veins are commonly affected by blood clots in severe COVID-19, with these events occurring in the lungs, heart, kidneys, and brain [25,26]. Since increased L-FABP is a highly sensitive marker of hypoxia, potentially caused by micro clots [27], its levels are likely to increase in patients who could later develop severe illness [28]. Therefore, we believe that urine L-FABP examination could become an essential tool for initial screening to assess symptom severity during health crises, such as SARS-CoV-2 and similar pandemics, as part of healthcare and public health sector management.

Nevertheless, the present study has some limitations. First, this was a single-center study; therefore, the management of patients with COVID-19 may vary depending on the practices of individual institutions and countries. Second, this was a retrospective and observational study; consequently, the treatment regimens differed among patients. This protracted experience with COVID-19 has stimulated the emergence of a diverse array of treatment options, with mainstream treatment regimens undergoing continual evolution. The dynamic treatment landscape is further complicated by variability in treatment availability across healthcare institutions, necessitating a tailored approach that considers the specific COVID-19 variant [29], patient illness severity, and comorbidities. Third, although we discussed the cutoff values for L-FABP levels and the SOFA score based on their sensitivity and specificity, the actual implementation of the model should be determined in consideration of factors such as epidemic trends and the availability of medical resources. Moreover, while the usefulness of L-FABP and the SOFA score has been demonstrated in this study, further discussion is needed on how to manage patients deemed unlikely to progress to severe illness and how to determine the optimal cutoff values depending on the timing of an outbreak. Finally, this study enrolled patients admitted to our institution between January 29, 2020, and April 6, 2022. Thus, the patient cohort included vaccinated and unvaccinated individuals, because mRNA COVID-19 vaccines were only introduced in Japan in May 2021. Moreover, the variant strain of COVID-19 differed depending on the period of time.

In conclusion, a combination of L-FABP levels and SOFA scores can improve the efficiency and accuracy of pre-admission screening for patients with COVID-19, potentially reducing unnecessary hospital admissions and optimizing resource allocation. The non-invasive nature of the L-FABP test makes it a valuable tool in a variety of clinical settings. Although promising, further research is warranted to validate our findings and refine predictive models for enhanced patient management during the ongoing pandemic.

## Supporting information

S1 Fig A. A scatter plot of SOFA and L-FABP on admission with Spearman’s rank correlation coefficient. **B.** Results of univariable logistic regression to detect severe cases. C. Results of multivariable logistic regression to detect severe/moderate cases.
(PDF)

S1 Data (CSV)
