# Supplementary material for: A retrospective cohort study evaluating the predictive value of urinary L-FABP combined with the SOFA score for assessing COVID-19 severity
Source: PLoS One. 2025 Sep 11;20(9):e0331558. doi: 10.1371/journal.pone.0331558 (PMC12425294; doi:10.1371/journal.pone.0331558)
Supplement: S1 Fig — A scatter plot of SOFA and L-FABP on admission with Spearman’s rank correlation coefficient. B. Results of univariable logistic regression to detect severe cases. C. Results of multivariable logistic regression to detect severe/moderate cases. (PDF) [file pone.0331558.s001.pdf]

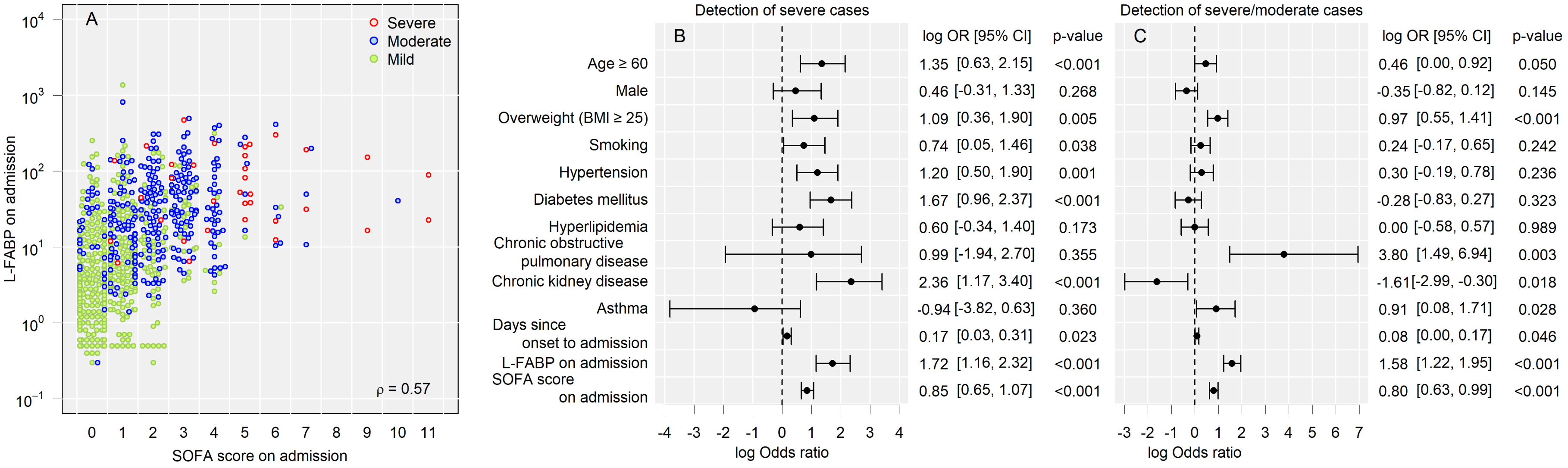

Supplementary Figure S1.

A. A scatter plot of SOFA and L-FABP on admission with Spearman’s rank correlation coefficient.

B. Results of univariable logistic regression to detect severe cases.

C. Results of multivariable logistic regression to detect severe/moderate cases.
